# Influences of large sets of environmental exposures on immune responses in healthy adult men

**DOI:** 10.1038/srep13367

**Published:** 2015-08-26

**Authors:** Buqing Yi, Marina Rykova, Gundula Jäger, Matthias Feuerecker, Marion Hörl, Sandra Matzel, Sergey Ponomarev, Galina Vassilieva, Igor Nichiporuk, Alexander Choukèr

**Affiliations:** 1 Hospital of the University of Munich (LMU), Department of Anesthesiology, Stress and Immunology Lab, Marchioninistrasse 15, 81377 Munich, Germany; 2 Institute of Biomedical Problems (IBMP), 123007 Moscow, Russia; 3 Max von Pettenkofer-Institut, University of Munich, Pettenkoferstraße 9a, 80336 Munich, Germany

## Abstract

Environmental factors have long been known to influence immune responses. In particular, clinical studies about the association between migration and increased risk of atopy/asthma have provided important information on the role of migration associated large sets of environmental exposures in the development of allergic diseases. However, investigations about environmental effects on immune responses are mostly limited in candidate environmental exposures, such as air pollution. The influences of large sets of environmental exposures on immune responses are still largely unknown. A simulated 520-d Mars mission provided an opportunity to investigate this topic. Six healthy males lived in a closed habitat simulating a spacecraft for 520 days. When they exited their “spacecraft” after the mission, the scenario was similar to that of migration, involving exposure to a new set of environmental pollutants and allergens. We measured multiple immune parameters with blood samples at chosen time points after the mission. At the early adaptation stage, highly enhanced cytokine responses were observed upon *ex vivo* antigen stimulations. For cell population frequencies, we found the subjects displayed increased neutrophils. These results may presumably represent the immune changes occurred in healthy humans when migrating, indicating that large sets of environmental exposures may trigger aberrant immune activity.

Globally increased migrant population is suspected to be related to an elevated incidence of allergic and autoimmune disease, and several clinical studies have provided statistical evidence for a special association between migration and increased risk of atopy/asthma^1^,^2^,^3^,^4^,^5^,^6^,^7^,^8^, suggesting an impact of migration related large sets of environmental exposures on the human immune system^9^. However, it is difficult to track retrospectively what kind of immune changes were displayed at the early stage following migration when the subjects were still healthy. Also, investigations about environmental effects on immune responses have been so far limited in candidate environmental exposures, such as air pollution^10^. For large sets of environmental exposures associated with a radical environmental change, for example, when one migrates, it is largely unknown to what extent this kind of environmental exposures can affect the human immunity in healthy adults^9^.

A simulated space mission to Mars-called Mars520 study-provided a rare chance to investigate this topic. The participants of the Mars mission lived in a simulated spacecraft for 520 days during a real interplanetary spaceflight (though microgravity or space radiation effects were not simulated). The simulated spacecraft was a closed environment and all the environmental factors were strictly controlled. When the participants left the closed habitat after the 520-d mission finished, they were exposed to a different set of pollutants and allergens from both indoor and outdoor environments, such as house dust mite and pollen. This scenario displays many similarities to that of migration.

In the current study, we investigated immune alterations occurred when the subjects left the closed environment after the 520-d mission. Cytokine response assays following *ex-vivo* fungal or bacterial antigen stimulation were performed to analyze immune activity changes.

## Results

### Immune responses to fungal or bacterial antigen stimulation

**Figure 1:**
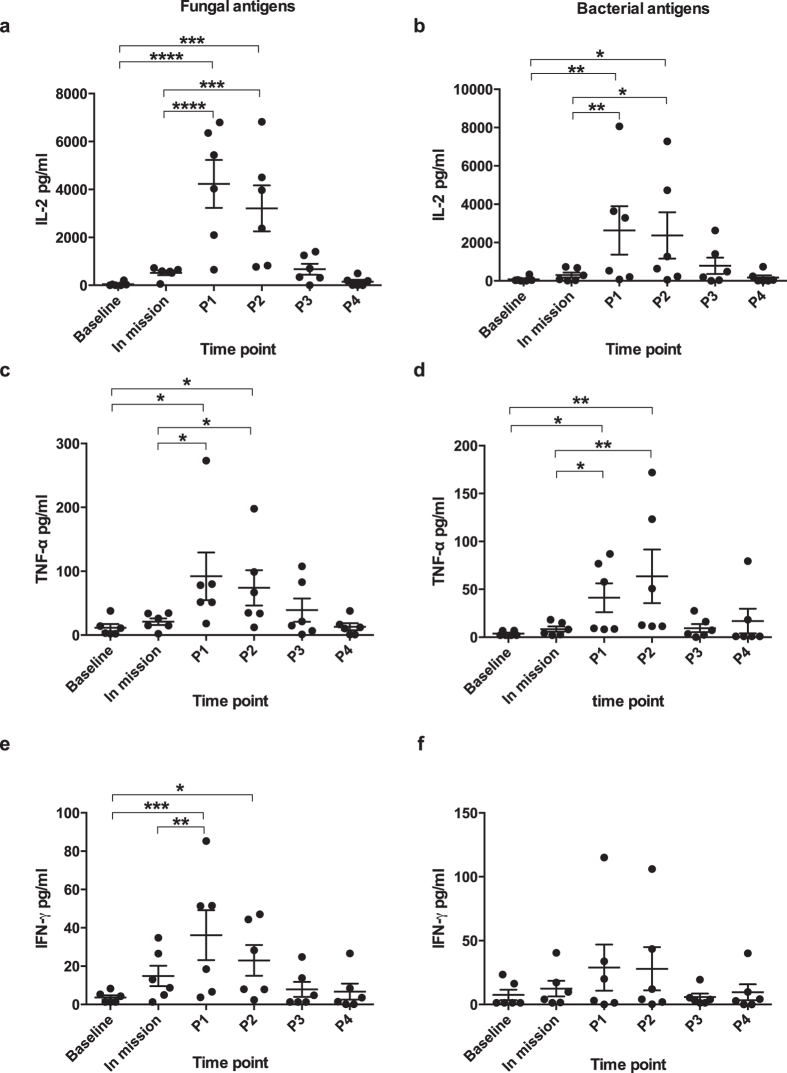
Cytokine responses to *ex vivo* fungal (a,c and e) or bacterial (b,d and f) antigen stimulation were highly enhanced after the subjects left the closed environment. For each time point, cytokine levels are presented with single subject data. Bars indicate mean ± SEM. Baseline samples were collected 7 days before the mission started. The time point “In mission” shows a representative time point before the end of the mission (day-510 in mission). P1, P2, P3 and P4 represent four post-mission time points after the subjects left the spacecraft: P1, 7 days post-mission; P2, 14 days post-mission; P3, 30 days post-mission; P4, 180 days post-mission. In response to both fungal and bacterial antigens, the productions of IL-2 and TNF-α were highly increased on P1 and P2 compared to baseline or “In mission”. Cytokine responses on day-510 (In mission), P3 or P4 showed no significant difference from baseline. *indicate a significant change (*P* < 0.05); **P ≤ 0.01; ***P ≤ 0.001; ****P ≤ 0.0001.

We assayed all the participants for proinflammatory responses against mixed fungal or bacterial antigens, as defined by the ability to up-regulate three representative proinflammatory cytokines IL-2, TNF-α and IFN-γ. The time points presented in this study were defined mainly based on the organizational schedule. In response to fungal antigens, on P1 the productions of IL-2, TNF-α and IFN-γ were all significantly higher than baseline (IL-2: *P* < 0.0001; TNF-α: *P* < 0.05; IFN- γ: *P* < 0.001) (Fig. 1). And the high productions were also observed on P2 (IL-2: *P* < 0.001; TNF-α: *P* < 0.05; IFN- γ: *P* < 0.05). In response to mixed bacterial antigens, on P1 the productions of IL-2, TNF-α were highly enhanced compared with baseline (IL-2: *P* < 0.01; TNF-α: *P* < 0.05). The high cytokine responses were also observed on P2 (IL-2: *P* < 0.05; TNF-α: *P* < 0.01). We did not observe significant increase of immune responses following bacterial or fungal antigen stimulation during the mission, as indicated by the results from the representative time point day-510 (labelled as “In mission”). In non-stimulated control samples, no changes of IL-2, TNF-α and IFN-γ were detected throughout the study interval (data not shown).

### Leukocyte distribution pattern was changed with increased neutrophils

The immunologic profiles of the subjects during the study period are shown in Table 1. Leukocyte distribution pattern displayed significant changes after the mission. On P1 and P2, the neutrophil percentages were increased compared with the baseline level (BDC) (*P*_BDC versus P1_ < 0.01; *P*_BDC versus P2_ < 0.05) or compared with that on day-510 (In mission) (*P*_In mission versus P1_ < 0.001; *P*_In mission versus P2_ < 0.01). Accordingly, the percentage of lymphocytes was reduced, which was mainly due to the increase of neutrophil numbers.

### Plasma cytokine level

To detect if there was systemic inflammation, we also measured circulating cytokines IL-2, TNF-α, IFN-γ in plasma samples. The results showed no significant increase of these proinflammatory cytokines after the subjects left the closed habitat (Table 2), indicating the increased immune responses were not brought about by systemic inflammation, which was also consistent with medical records that no symptoms of infection were detected during the study period.

### Stress hormone cortisol levels were close to the baseline

**Figure 2:**
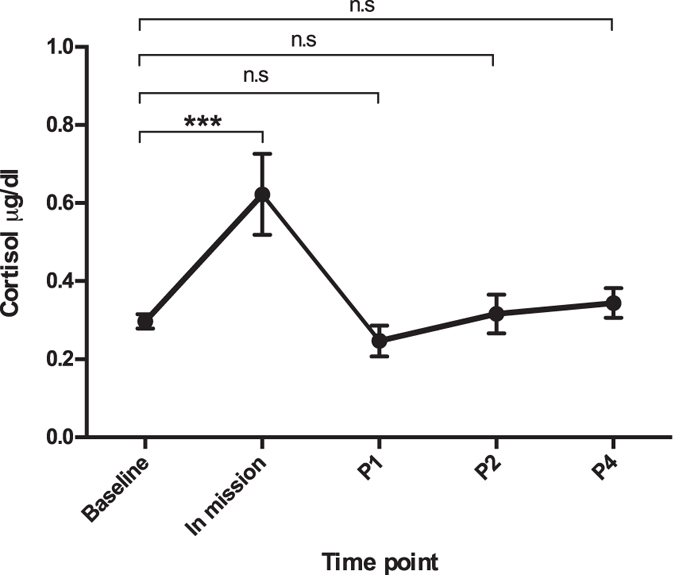
Salivary cortisol. Values show mean ± SEM. On P1, P2 and P4, the cortisol levels showed no difference from baseline. At the time point day-510 (In mission), the cortisol levels were significantly higher than baseline.

As shown in Fig. 2, although the subjects displayed high cortisol levels during the mission, cortisol levels were close to the baseline at all the post-mission time points (P1, P2 and P4) suggesting low/no stress conditions after the subjects left the closed habitat.

## Discussion

In the current study, we performed a variety of immune analyses to investigate immune alterations when the subjects were exposed to a different environment after they finished their long-duration simulated space mission. Results of this study indicated that the subjects displayed aberrant immune activity of an overshoot of proinflammatory cytokines in response to fungal or bacterial antigen stimulation, and increased neutrophils were observed. During the study period, no systemic inflammation or significant changes of stress hormone cortisol were detected, suggesting the hypersensitive immune responses were not brought about by these *in vivo* factors.

In contrast, we previously reported that during the 520-d mission period, cortisol levels were elevated and the participants showed increased lymphocyte numbers^11^. Taken into consideration of the distinct cortisol levels and contrasting leukocyte distribution patterns displayed “during” versus “after” the mission, we assume the aberrant immune activity and neutrophil increase observed post-mission are based on a different mechanism from that of the neuroendocrine and immune changes occurred during this simulated space mission.

Interestingly, neutrophil increase was observed consistently after spaceflight^12^,^13^. Previously it was assumed that microgravity, radiation and the landing process from spaceflight resulted in the increase of neutrophil. It was unexpected that the participants in this simulated space mission also displayed increased neutrophils since no microgravity, radiation and landing process were simulated. However, the participants of this simulated Mars mission and real spaceflights were all exposed to a different environment after their mission finished. Therefore, the influence of large sets of environmental exposures, at least partly, should have contributed to the neutrophil increase in the current study. So far, no similar cytokine response analyses have been reported after spaceflight, but immune overreactions such as increased allergy symptoms could be frequently found in the anecdotal accounts of the astronauts´ health after spaceflight^14^.

Environmental factors have long been known to be able to affect immune activity from both animal studies and human studies^6^,^15^,^16^,^17^,^18^. Over the past few decades many efforts have been made to understand the interaction between various environmental factors, genetic factors and the development of allergic disease^19^,^20^,^21^,^22^,^23^. In particular, multiple migration studies have provided important information on the role of environmental factors in the development of atopy and asthma, indicating immigrants, in general, are more prone to the development of allergies than the local population^24^,^25^,^26^,^27^. The immune alterations we observed in this special migration study – migration from space to earth - might have both protective and damaging roles, which can act as a host defensive strategy in a short term, but may also enhance vulnerability due to excessive immune responses like allergic host defenses^28^ and lead to allergic inflammation or other related immune diseases. These immune changes explain to certain extent the results of previous clinical studies^24^,^25^,^26^,^27^.

We can speculate about how environmental exposures may have contributed to the immune changes in the current study. Acute neutrophil increase can often be observed in the setting of systemic infection^29^. In our study, it was likely that the presence of large sets of environmental exposures was falsely sensed by the host-defense mechanism as a “threat” similar to systemic infection, and the immune system was accordingly alerted. Then this false emergency state was translated into a series of molecular events that stimulate enhanced neutrophil production to combat the “systemic” threat. As reported by other studies^30^,^31^,^32^, the powerful but relatively low target specificity of neutrophils may result in hyperactive immune responses by reducing activation threshold of T helper cells. This might explain our observations in this study that along with the increase of neutrophil numbers, cytokine responses were significantly amplified against both fungal and bacterial antigen stimulations. Further investigation with animal model is needed to understand the underlying mechanism of these immune changes.

In summary, in this study the participants underwent noticeable immune changes and displayed hypersensitive immune responses when they were exposed to a different environment. These results may presumably represent the immune changes occurred in healthy migrants and it is likely that such aberrant immune activity might have potential of paving the way for allergy development in the future. Preventive strategies should be taken into consideration for highly susceptible ones when they are exposed to a sudden environmental change, such as following migration. The information we have acquired from this study adds to evidence about the effects of environmental exposures on immune responses in healthy adults, and might be able to provide insights for the underlying mechanism of the special association between migrants and risk of allergic diseases.

## Methods

### Participants and environmental conditions

The Mars520 spaceflight simulation study was conducted at the Institute of Biomedical Problems (IBMP) in Moscow and approved by ethical boards of the Russian Federation and the European Space Agency. The ethical committee of the Hospital of the University of Munich granted approval for the investigation presented in this report (Approval Nr.: 222-08). Six healthy male volunteers were selected based on modified astronaut selection criteria (mean ± SD; age(y): 33 ± 6; size (m): 1.76 ± 0.04; weight (kg): 81 ± 5; BMI: 26 ± 2). They provided written informed consent after due approval to spend 520 days in an enclosed habitat consisting of hermetically sealed interconnecting modules simulating a real spaceship. All studies were done as outlined in the Declaration of Helsinki. All participants underwent a thorough clinical examination prior to participation.

Similar to real spaceflight, environmental factors were maintained constant in the simulated spaceship. Registration of the environmental parameters was conducted every two hours. In the enclosed habitat, temperature and optimal relative humidity were automatically maintained in the given range. Microbial contamination level was regularly controlled (not less than once a month) in compliance with the requirements of the standard for spaceship. More detailed information about this mission has been reported elsewhere^11^.

### Blood Sampling

Blood samples were drawn into blood tubes containing either EDTA or heparin as anticoagulants between 7 and 8 AM in the morning. *Ex vivo* simulated fungal and bacterial infection assay, differential blood count and immunophenotype analysis, were performed within one hour after blood drawing. Plasma samples were immediately frozen at -80°C. Baseline Data (BDC) samples were collected 7 days before the mission started. After the 520-d mission finished, samples were collected 7 days post-mission (indicated as time point P1), 14 days post-mission (indicated as time point P2), 30 days post-mission (indicated as time point P3) and 180 days after the mission (indicated as time point P4), respectively. Owing to operational difficulty, immune phenotype analysis was not performed at the time point P3. In addition, to show a representative condition before the crew exited their habitat, results from day-510 (10 days before the mission finished) were presented (labelled as “In mission”).

### Saliva collection

The Salivettes® sampling device (Sarstedt, Nümbrecht, Germany) was used to collect saliva samples immediately after awaking (between 7 to 8 a.m.). Likewise, owing to operational reason, no saliva samples were collected at the time point P3.

### *Ex vivo* fungal or bacterial antigen stimulation assay

A fungal antigen mixture containing Candida-lysate (10 μg/ml, Allergopharma, Reinbeck, Germany) and Trichophyton-lysate (10 μg/ml, Allergopharma, Reinbeck, Germany) was used for fungal antigen stimulation. A bacterial antigen mixture containing Diphterie-, Tetanus- and Pertussis-toxoid (all three combined in 1% Boostrix®, GlaxoSmithKline, Munich, Germany) was used for bacterial antigen stimulation. Both assays were performed within one hour after blood drawing using a previously described method^33^ with small modification. The assay tubes were incubated for 48h at 37 °C, and then the supernatant was transferred into an Eppendorf tube and immediately frozen at –80 °C for further measurements.

### Differential blood count

Absolute white blood cell count and the percentage of each type of white blood cell were measured with whole blood samples immediately after the specimen collection using the Celltac alpha MEK 6318 type full automatic Hematology Analyzer (Nihon Kohden, Japan).

### Immunophenotype Analysis

Peripheral blood immunophenotype analysis was performed with flow cytometry using the following antibodies (IQ PRODUCTS, Netherlands): CD3, CD4, CD8, CD19, CD16 & CD56. T lymphocytes, B lymphocytes, helper/inducer T lymphocytes, suppressor/cytotoxic T lymphocytes, and natural killer (NK) lymphocytes were identified. The blood samples were processed as described in the manual. The stained leukocytes were analyzed on the FACSCalibur flow cytometer (Becton Dickinson, USA) using CellQuest software for data collection and analysis. 15,000 events were analyzed per tube. Isotypic controls were used for each assay to determine nonspecific staining. The fluorescence compensation was performed using CaliBRITE beads (Becton Dickinson, USA) and FACSComp software.

### Cytokine measurement

Cytokines were measured in a blinded fashion using LuminexxMAP® technology (Bioplex®) with commercially available reagents from BioRad-Laboratories Inc. (California, USA) according to the manufacturer´s guidelines. All the samples were batch analyzed. Data were analyzed with Bioplex®-Software, and assay sensitivities for cytokines interleukin-2 (IL-2), interferon-γ (IFN-γ) and tumor-necrosis-factor (TNF)–α was 1.6, 6.4 and 6.0 pg/ml, respectively.

### Cortisol analysis

Salivary cortisol was quantified with an automated immunoassay system based on the principle of electro-chemiluminescence (Elecsys 2010, Roche, Mannheim, Germany) at the Institute of Clinical Chemistry, Hospital of the University of Munich, Germany.

### Statistical methods

All data were tested for normal distribution using the Shapiro-Wilk test. Changes of each parameter across the time points were compared using Repeated-Measure Analyses of Variance. Post-hoc tests comparing collection time points of day-510, P1, P2, P3, P4 with baseline (or with day-510) were performed. A P-value < 0.05 was regarded as statistically significant. SigmaPlot 12.0 (Systat Software, Chicago, Illinois, USA) was used for data analyses.

## Additional Information

**How to cite this article**: Yi, B. *et al.* Influences of large sets of environmental exposures on immune responses in healthy adult men. *Sci. Rep.* **5**, 13367; doi: 10.1038/srep13367 (2015).

## Figures and Tables

**Table 1:** Peripheral leukocyte distribution pattern.

	Baseline	In mission	P1	P2	P3	P4
	Mean ± SE	Mean ± SE	Mean ± SE	Mean ± SE	Mean ± SE	Mean ± SE
Leukocytes§	4.54 ± 0.23	4.60 ± 0.38	4.90 ± 0.48	4.54 ± 0.31	n/a	4.88 ± 0.21
Neutrophils						
% Leukocytes	50.31 ± 2.15	47.37 ± 2.33	57.92** ± 2.14	57.20* ± 3.25	n/a	47.78 ± 1.44
Absolute count§	2.30 ± 0.20	2.15 ± 0.14	2.85** ± 0.35	2.59* ± 0.23	n/a	2.32 ± 0.07
Lymphocytes						
% Leukocytes	42.92 ± 3.08	48.51* ± 2.02	37.90* ± 1.95	38.73 ± 3.87	n/a	41.73 ± 0.97
Absolute count§	1.93 ± 0.13	2.25 ± 0.26	1.82 ± 0.15	1.60 ± 0.18	n/a	2.05 ± 0.11
CD19+ B cells§	0.25 ± 0.04	0.27 ± 0.04	0.23 ± 0.03	0.20 ± 0.04	n/a	0.26 ± 0.04
CD3+ T cells§	1.41 ± 0.07	1.55 ± 0.17	1.25 ± 0.10	1.09 ± 0.11	n/a	1.43 ± 0.18
CD3^+^CD8^+^ T cells§	0.37 ± 0.02	0.41 ± 0.03	0.32 ± 0.03	0.28 ± 0.03	n/a	0.41** ± **0.08
CD3^+^CD4^+^ T cells§	0.88 ± 0.07	0.97 ± 0.15	0.74 ± 0.07	0.69 ± 0.10	n/a	0.93 ± 0.16
NK cells§	0.21 ± 0.04	0.24 ± 0.06	0.21 ± 0.05	0.16 ± 0.04	n/a	0.23 ± 0.07

Baseline samples were collected 7 days before the mission. P1, P2, P3&P4 represent time point 7 days post-mission (P1), 14 days post-mission (P2), 30 days post-mission (P3) and 180 days post-mission (P4), respectively. The time point “In mission” shows a representative time point before the end of the mission (day-510 in mission).

n/a, data not available. *indicates a significant change (*P* < 0.05) to baseline; **P ≤ 0.01.

^§^Absolute cell counts are × 10^3^/μL.

**Table 2:** Summary of plasma cytokine levels.

Cytokine concentraion (pg/ml)	Baseline	In mission	P1	P2	P3	P4
	Mean ± SE	Mean ± SE	Mean ± SE	Mean ± SE	Mean ± SE	Mean ± SE
IL-2	1.67 ± 1.63	0.22 ± 0.15	1.98 ± 0.58	1.83 ± 1.68	1.41 ± 0.59	1.16 ± 0.66
IFN-γ	9.49 ± 1.40	6.37 ± 0.23	12.51 ± 6.14	8.45 ± 1.32	12.62 ± 5.10	13.02 ± 4.33
TNF-α	2.37 ± 0.40	1.85 ± 0.32	3.52 ± 1.33	2.26 ± 0.26	2.69 ± 0.52	1.86 ± 0.44

No significant differences between the indicated time point and baseline.
